# An exploratory analysis of 5‐alpha reductase inhibitors and risk of opioid use disorder among male Medicare beneficiaries receiving prescription opioid medications

**DOI:** 10.1111/ajad.70069

**Published:** 2025-07-13

**Authors:** Abdelrahman G. Tawfik, Tyler Lister, Ainhoa Gomez‐Lumbreras, Randall T. Peterson, Marco Bortolato, Daniel C. Malone

**Affiliations:** ^1^ Department of Pharmacotherapy, College of Pharmacy University of Utah Salt Lake City Utah USA; ^2^ Department of Pharmacology and Toxicology, College of Pharmacy University of Utah Salt Lake City Utah USA; ^3^ Department of Pharmacodynamics, College of Pharmacy University of Florida Gainsville Florida USA

## Abstract

**Background:**

Opioid use disorder (OUD) imposes a significant socioeconomic burden, highlighting the need for new interventions.

**Objective:**

This study investigated whether exposure to 5‐alpha reductase inhibitors (5αRIs) is associated with a lower risk of developing OUD.

**Methods:**

A cohort study was conducted using Medicare data from 2017 to 2019. Male subjects identified as receiving at least one opioid medication were propensity score matched based on exposure to a 5αRI medication. The primary outcome of interest was a diagnosis of OUD occurring following opioid exposure. Additionally, the study compared the number of morphine milliequivalents (MME) and the count of opioid prescription claims between the groups.

**Results:**

We identified 467,399 subjects who had received at least one opioid prescription. Among these, 19,176 beneficiaries were receiving a 5αRI before opioid medication exposure and were matched 1:1 to non‐users. Use of 5αRI was associated with a reduced risk of OUD (OR = 0.63, 95% CI: 0.53–0.75). MME for subjects exposed to 5αRI was significantly lower than for those without 5αRI exposure (*p* < .001). Furthermore, there was a significant difference in the number of opioid prescription claims between the groups, with those taking a 5αRI having an average of 6.0 (SD = 10.1) prescriptions as compared to 6.7 (SD = 13.0) for those not receiving a 5αRI (*p* < .001).

**Conclusions and Scientific Significance:**

This first of its kind in humans study suggests that concomitant use of opioids and 5αRI is associated with a reduction in OUD and maybe a preventive therapy for patients at risk of OUD. Our findings align with animal models that have shown similar findings.

## INTRODUCTION

Opioid use disorder (OUD) is a chronic disease characterized by a persistent and overpowering desire to use opioids despite significant substance‐related problems. These issues include recurring difficulties in social and occupational spheres due to opioid consumption, increased tolerance, and withdrawal syndrome upon discontinuation.^1^, ^2^ Mitigating the risk of developing OUD is critical, as up to 35% of chronic opioid users have been shown to develop OUD.^3^ Efforts have to be made to prevent OUD in patients undergoing such treatments.^4^ Currently available OUD treatments include behavioral therapy, counseling, and medication‐assisted treatment with buprenorphine, naloxone, and/or methadone.^5^ Despite these options, high rates of relapse and opioid‐related mortality persist, underscoring the urgent need for novel interventions.

Recent investigations in animal models suggest that opioid seeking may be mitigated by 5‐alpha reductase inhibitors (5αRIs), such as finasteride and dutasteride, which are clinically approved for therapeutic use in benign prostatic hyperplasia (BPH) and androgenetic alopecia.^6^ The enzyme 5α‐reductase converts testosterone to 5α‐dihydrotestosterone (DHT), a potent androgen influencing sexual differentiation in males. The primary action of 5αRIs is to decrease DHT synthesis, which contributes to their therapeutic effects. While the specific mechanism whereby 5αRIs decrease opioid self‐administration in animal models remains unclear, it is likely based on alterations of a different metabolic pathway, namely the biosynthesis of neurosteroids in the brain.^7^ These molecules play a critical role in the modulation of anxiety, mood, and impulse control,^8^, ^9^ all of which are relevant to opioid misuse. In keeping with this idea, 5αRIs have been shown to influence these behavioral domains in humans and animals.^10^, ^11^, ^12^, ^13^, ^14^ 5αRIs, including finasteride and dutasteride, are FDA‐approved for treating BPH, a common condition in aging men characterized by urinary tract symptoms. These medications are typically prescribed to men over the age of 50 and are also used off‐label in lower doses for androgenetic alopecia. The primary therapeutic mechanism involves the reduction of DHT levels, which contributes to prostate volume reduction.

Notably, animal studies indicate that, while these drugs may reduce the rewarding effects of natural and synthetic opioids, they do not impact their analgesic properties.^7^ This premise provides the basis for this study to determine if concomitant treatment with 5αRI and opioids is associated with a lower incidence of OUD in humans. This study was considered a hypothesis‐generating study to determine if there is evidence to justify further clinical research using more controlled study designs. In addition to assessing the risk of OUD, we also examined total opioid exposure, measured through morphine milligram equivalents (MMEs) and prescription count, as secondary outcomes to explore whether 5αRI use is associated with reduced opioid intensity, which may underlie differences in OUD development. Therefore, the purpose of this study was to evaluate if concurrent use of 5αRIs and opioids may reduce the risk of OUD using an observational study of male Medicare beneficiaries.

## MATERIALS AND METHODS

This was an observational cohort study, using data from Medicare covering the years 2017 to 2019. This manuscript follows the RECORD‐PE guidelines for reporting pharmacoepidemiological research undertaken using routinely collected data.^15^

### Setting and participants

Medicare beneficiaries enrolled in Parts A (inpatient), B (physician services), and D (outpatient medications) were eligible for inclusions. Individuals of recorded male gender receiving at least one opioid prescription, with at least 6 months of observational time, were eligible for inclusion. Subjects were excluded if they had a diagnosis of hospice care, had only received parenteral opioid medications, used cough or cold products containing opioids, had a diagnosis of malignant tumors, or were enrolled in Medicare Advantage plans. Additionally, individuals who died before 6 months of observation time were excluded from the study.

### Drug exposure

Exposure to an opioid was determined through pharmacy claims, identifying individuals who had been prescribed at least one opioid medication. Both single‐entity and combination products containing opioids were used to identify the cohort. For each opioid prescription claim, we calculated the total amount of MMEs based on the specific opioid and its strength.^16^ We also calculated the total number of opioid prescription claims over the entire study period for each individual. 5αRI exposure was defined in individuals receiving at least one prescription of a 5αRI before exposure to an opioid medication. Given the chronic nature of 5αRI treatment, the analysis did not exclude individuals based on the duration of the 5αRI use. Thus, this study did not examine new incident users of 5αRI treatment but did require the diagnosis of OUD (see below) to be after an opioid prescription claim. While 5αRI exposure was required to precede opioid use, and OUD diagnosis to occur after opioid exposure, we did not apply a washout period for prior opioid or OUD exposure, which limits our ability to confirm incident use. Therefore, it is possible that some individuals included in the analysis had pre‐existing OUD.

### Independent variables

Sociodemographic (age, sex, race, geographic region, and marital status), comorbidities related to mental and behavioral conditions (considered predictors for a higher probability of OUD), prostate cancer (ICD‐10 code diagnosis: C61), and diagnoses in the Charlson Comorbidity Index (CCI) were included.^17^, ^18^, ^19^ Conditions included in the CCI are: myocardial infarction; congestive heart failure; peripheral vascular disease; history of cerebrovascular accident; dementia; chronic obstructive pulmonary disease; connective tissue disease; peptic ulcer disease, liver disease; diabetes; hemiplegia; solid tumor; leukemia; lymphoma; and acquired immune deficiency disease.

### Outcomes of interest

The primary outcome of interest was a diagnosis of opioid misuse based on the International Classification of Disease Tenth Edition (ICD‐10th), listed as a primary diagnosis of all F11.xx ICD‐10 codes.^19^ See the supplementary materials for the frequency of diagnosis codes for the eligible cohort. While other potential diagnosis codes are associated with opioid misuse (e.g., opioid poisoning, intoxication, etc.), we did not rely on those diagnoses because they may not reflect addiction to opioids, the condition that may be affected by 5αRIs. Secondary outcomes of interest include the MME for opioid prescription claims and the total number of opioid prescriptions.

### Statistical methods

Due to issues of confounding by indication, we conducted a matched propensity score matching (PSM) analysis using both a 1:1 Greedy nearest neighbor match and an inverse probability of treatment weighting (IPTW). A propensity score was estimated using age, race, comorbidities, CCI, history of mental and/or behavioral diagnosis, and a diagnosis of prostate cancer. Standardized mean differences between the matched cohorts were calculated.

Descriptive analyses were conducted using parametric (Student's *t*‐test and correlation) and non‐parametric (Chi‐square) statistical procedures. The primary analysis examined the incidence of OUD for the 1:1 PSM samples. Unadjusted, adjusted, and IPTW logistic regression analyses were also conducted. The adjusted logistic regression included the independent variables of 5αRI use, age, race, CCI, diagnosis of prostate cancer, and history of mental and behavioral disorders. Comparisons between users and non‐users of 5αRI were made with respect to the MME per prescription, total number of MME, and number of opioid prescriptions obtained over the duration of the study. SAS version 9.4 (SAS Institute Inc.) statistical package was used to conduct all the statistical analyses. Conventional type I error rate of 0.05 was used to determine statistical significance.

## RESULTS

### Cohort characteristics

The study included a total of 467,399 males from Medicare's nationwide database who were all prescribed at least one opioid and met all the other inclusion and exclusion criteria. Among these individuals, 19,176 (4.1%) were receiving a 5αRI, and 13,716 (2.9%) had a diagnosis of OUD. Table 1 displays the distribution of the cohorts before and after PSM, including the presence of mental health conditions. After matching, it was observed that the 5αRI users had a higher prevalence of history of acute myocardial infarction (*p* = .04) and osteoporosis (*p* = .01). Also, diagnoses of prostate cancer were observed more frequently among non‐users (7.4%) compared to 5αRI users (5.4%) (*p* < .001). However, the overall comparison of the matched cohorts showed them to be similar in terms of demographics, including presence of mental health conditions, with standardized mean differences of less than 0.2 across all characteristics.

**Table 1:** Demographic characteristics of beneficiaries using opioid medications before and after propensity score matching.

	Before matching			After 1:1 Greedy matching			
Characteristic	5αRI Use *N* = 19,176	No 5αRI Use *N* = 453,683	*p*‐Value^a^	5αRI Use *N* = 19,176	No 5αRI Use *N* = 19,176	*p*‐Value^a^	Standardized difference
Age (mean, Std Dev)	76.1 (8.3)	68.7 (12.1)	**<.001**	76.1 (8.3)	76.1 (8.4)	.86	0.07
Race *n* (%)							
White	16,723 (87.2)	376,468 (84.0)	**<.001**	16,723 (87.2)	16,827 (87.8)	.09	
Black	1003 (5.2)	37,519 (8.4)		1003 (5.2)	911 (4.8)		
Other	1450 (7.6)	34,236 (7.4)		1450 (7.6)	1438 (7.5)		
Charlson Comorbidity Index Score	1.07 (1.6)	1.0 (1.6)	**<.001**	1.07 (1.6)	1.05 (1.6)	.20	0.013
**Comorbidities *n* (%)**							
Alzheimer disease	1,424 (7.4)	16,873 (3.8)	**<.001**	1424 (7.4)	1367 (7.1)	.26	−0.013
History of acute myocardial infarction	416 (2.2)	8586 (1.9)	**.01**	416 (2.2)	360 (1.9)	.04	−0.021
COPD	4144 (21.6)	81,054 (18.1)	**<.001**	4144 (21.6)	4087 (21.3)	.48	−0.090
Kidney disease	5 (0.03)	1155 (0.3)	**<.001**	5 (0.03)	2 (0.01)	.25	0.048
Depression	4768 (24.9)	103,696 (23.1)	**<.001**	4768 (24.9)	4742 (24.7)	.76	−0.041
Diabetes	7882 (41.1)	171708 (38.3)	**<.001**	7882 (41.1)	7903 (41.2)	.83	0.002
Hip fracture	313 (1.6)	3810 (0.9)	**<.001**	313 (1.6)	275 (1.4)	.11	−0.018
Osteoporosis	717 (3.7)	10851 (2.4)	**<.001**	717 (3.7)	624 (3.3)	.01	−0.075
Rheumatoid and osteoarthritis	11,312 (59.0)	226,400 (50.5)	**<.001**	11,312 (59.0)	11,276 (58.8)	.71	−0.003
Mental health diagnosis	6615 (34.0)	167,430 (37.3)	**<.001**	6615 (34.0)	6529 (34.1)	.88	0.002
Prostate cancer	1033 (5.4)	27,104 (6.1)	**.0002**	1033 (5.4)	1429 (7.5)	<.001	0.10

Abbreviations: COPD, chronic obstructive pulmonary disease; Std dev, Standard deviation.

^a^ Bolded values indicate statistical significance at a Type I error rate of 0.05.

### Analyses of concurrent OUD and 5αRI usage

Results examining the outcome of OUD are shown in Table 2. The unadjusted risk of OUD across the entire sample was 0.37 (95% CI: 0.32–0.42). For the PSM 1:1 matched cohorts, the odds of OUD were 0.63 (95% CI: 0.53–0.75). The adjusted logistic regression model, applied to these matched cohorts, found a slightly greater protective effect of 5αRIs (OR = 0.58, 95% CI: 0.51–0.69). Similar results were obtained using the IPTW propensity method (see Table 2).

**Table 2:** Logistic regression predicting opioid use disorder among male medicare beneficiaries with‐ and without prior exposure to 5‐alpha reductase inhibitors.

Method	Odds ratio^b^	Lower 95% bound	Upper 95% bound
Unadjusted (full cohort)	**0.37**	0.32	0.42
Logistic regression^a^ (full cohort)	**0.58**	0.51	0.69
Propensity score matched 1:1	**0.63**	0.53	0.75
Inverse probability weighting propensity score matched	**0.56**	0.54	0.58

a Adjusted for age, race, Charlson Comorbidity Index score, mental health diagnosis, prostate cancer diagnosis.

^b^ Bolded values indicate statistical significance with Type 1 error rate of 0.05.

Exposure to opioid medications among both users and non‐users of 5αRIs is shown in Table 3. The MMEs per prescription claim for non‐users of 5αRIs was 106.7 MME (SD = 427.7) as compared to 85.5 MME (SD = 292.3) for users of 5αRIs (*p* < .001). The total MME obtained over the entire study period was also significantly lower for those beneficiaries exposed to 5αRIs as compared to individuals not receiving 5αRIs (*p* < .001). Furthermore, the number of opioid prescription claims individuals received was lower among 5αRI users (mean = 6.0, SD = 10.1) as compared to non‐users (mean = 6.7, SD = 13.0) (*p* < .001).

**Table 3:** Morphine equivalent doses and prescriptions for medicare beneficiaries with and without prior exposure to 5‐alpha reductase inhibitors.

	5αRI Use	No 5αRI Use	
Characteristic	*N* = 19,176	*N* = 19,176	*p*‐Value^a^
MME per prescription	85.5 (SD = 292.3)	106.7 (428.7)	**<.001**
Total MME per beneficiary	5110.8 (SD = 22,387.4)	11,104.9 (SD = 37,572.3)	**<.001**
Number of opioid prescription claims	6.0 (SD = 10.1)	6.7 (SD = 13.0)	**<.001**

Abbreviation: MME, morphine milliequivalents.

^a^ Bolded values indicate statistical significance differences between groups with Type I error rate of 0.05.

## DISCUSSION

The results of this study were that individuals who simultaneously use opioids and a 5αRI, such as finasteride, had significantly reduced odds of being diagnosed with OUD compared to those not using 5αRIs. This effect was consistent across a variety of analytical methods applied to evaluate observational data, including two different approaches to PSM as well as conventional logistic regression models. In addition, persons receiving a 5αRI consumed fewer opioid medications over the study period, and also when they did consume opioids, the total number of MME was fewer. Overall, these findings are in substantial alignment with previous evidence on zebrafish and rat models, indicating that 5αRIs reduced the self‐administration of various opioids.^6^ This epidemiological evidence strengthens the hypothesis that 5αRIs might be used as preventive or therapeutic tools in OUD management. Notably, evidence from rat models suggests that 5αRIs do not diminish,^6^ and may even enhance,^20^ the analgesic and antinociceptive effects of opioids. This observation opens the possibility that 5αRIs could be used in association with opioids to lessen the risk of misuse in patients receiving opioids as pain medications. Such a combination is predicted to maintain the effectiveness of opioid analgesia while potentially reducing the liability for misuse and development of OUD. These promising findings underscore the need for future prospective studies to verify whether 5αRIs can effectively mitigate OUD risk and symptom severity. If such trials demonstrate consistent results, 5αRIs may hold potential as preventive or therapeutic agents in the management of OUD.

This prospective research would be crucial in confirming the therapeutic potential of 5αRIs in OUD treatment and prevention.

The exact mechanism by which 5αRIs reduce opioid self‐administration is not entirely clear, but several theories may be proposed to explain these effects. One possibility involves the reduced conversion of testosterone to DHT. Given the modulatory role of androgens in the behavioral effects of opioids, an imbalance between these two androgens could influence opioid seeking.^21^ Alternatively, a significant mechanism might be the inhibition of neuroactive steroid biosynthesis and metabolism.^17^ For instance, finasteride was shown to alter the levels of neuroactive steroids like dehydroepiandrosterone (DHEA) and its sulfate metabolite (DHEAS) in zebrafish brains.^5^ These steroids are implicated in the prevention of morphine dependence in animal studies.^22^ Furthermore, DHEA was shown to be effective in reducing withdrawal symptoms in a subgroup of heroin addicts with no previous use of other substances of abuse.^23^ Another potential mechanism of action involves the reduced synthesis of the neurosteroid allopregnanolone, which regulates anxiety, mood, and stress responses through its action on GABA‐A receptors in the brain. However, it should be noted that this study controlled for anxiety and depression, which have been identified as a risk for OUD.^17^ In particular, we included the presence of a diagnosis for mental and behavioral health conditions that aligns with previously published on OUD.^17^, ^24^ Overall, this reduces the possibility that the observed effects may represent behavioral alterations related to other mental disorders.

Irrespective of the specific mechanisms supporting the properties of OUD, it is worth noting that 5αRIs have been shown to reduce impulsivity across several constructs, including delay and probability discounting.^12^, ^13^ Given the essential role of impulsivity in shaping the propensity to engage in misuse of opioids and other drugs, these results point to the possibility that 5αRIs may reduce abuse and dependence liability for a wide range of substances. In line with this idea, previous studies suggest that finasteride reduces alcohol drinking in men and mice.^10^, ^25^ Furthermore, this drug interferes with the behavioral outcomes of dopamine receptor activation in the ventral striatum and prefrontal cortex,^16^ which is central to the development of substance use disorders. Furthermore, finasteride was also shown to oppose the propensity for pathological gambling, a behavioral addiction, in Parkinson's disease patients treated with dopaminergic agonists.^9^ Future epidemiological studies are warranted to study whether 5αRIs are associated with a lower odds ratio for other substance use disorders. In particular, the possibility that 5αRIs may be used to reduce the propensity to risky behaviors by interfering with dopaminergic signaling in corticolimbic circuits suggests that these drugs may be incorporated into novel proactive strategies to mitigate the risk of addictions in predisposed individuals.

## LIMITATIONS

The average age of our participants was 76.1 ± 8.3. Thus, the present study does not offer sufficient insight into the effects of 5αRIs in reducing OUD risk in younger men, who display a higher risk for this disorder. Previous observational studies of 5αRI have included mainly men over 65 years of age.^26^, ^27^ A concern related to using 5αRI for OUD is the possibility of adverse events. Short‐term use of 5αRI agents has been associated with an increased risk of incident dementia,^28^ and longer‐term use has been shown to increase the risk of heart failure.^29^, ^30^ It should also be noted that the use of finasteride, particularly in younger men, is associated with various adverse effects, including difficulty with sexual performance, diminished ability to concentrate, and decreased feelings of life pleasure or emotions.^31^, ^32^ Notably, these effects have been reported to persist even after 5αRI discontinuation.^33^ Other adverse effects of these agents exist as well.^34^ We only studied a male population, as indications for 5αRI are limited to males; however, these medications are used off‐label in females to treat alopecia or hirsutism.^35^ Because 5αRIs are predominantly prescribed to older males for BPH, our analysis was limited to male Medicare beneficiaries. Thus, the applicability of these findings to women, including those receiving 5αRIs off‐label, remains uncertain. Future studies must assess potential sex differences in the neurobiological response to 5αRIs and their impact on OUD risk.

Other key limitations should be kept in mind when interpreting the present results. As with all analyses of administrative data, miscoding, incomplete coding, and undercoding of diagnoses may affect the results. There is likely an underreporting of OUD due to stigma, but the degree of this bias is unknown. We also did not attempt to control other medications that might be associated with OUD, such as benzodiazepines,^36^ stimulant medications, or medications associated with treatment/reversal of OUD,^37^ such as buprenorphine and naloxone.

The lack of a washout period for prior opioid use or pre‐existing OUD is a limitation of this exploratory analysis. Prevalent users may differ in opioid response compared to incident users, potentially modifying their interaction with 5αRI treatment. Use of a new user design with adequate washout could lead to findings that are moderated toward the null, or consistent with the current results. Therefore, future research using incident new‐user designs is needed to validate and refine these findings.

We reported the results for total MME because this is a standard approach to representing the amount of opioid medications consumed, given that there are many various forms of opioids (e.g., codeine, oxycodone, hydrocodone, morphine, etc.). The observed differences in total MME and prescription counts between groups reflect opioid exposure, with those concurrently exposed to 5aRA using fewer opioid‐related medications that may lead to lower OUD abuse. However, they may also stem from underlying pain severity or treatment duration differences not captured in our data set. While propensity score matching adjusted for multiple clinical and behavioral characteristics, residual confounding by indication remains a limitation.

Another limitation is that 5αRIs are primarily used by older men for the treatment of BPH, which requires higher doses of these drugs (i.e., 5 mg/day of finasteride). Thus, we do not know whether dosages associated with the treatment of androgenic alopecia (1 mg/day of finasteride) may have equivalent effects on reducing OUD risk. In addition, because we required at least 6 months of enrollment, there is the possibility of immortal time bias. However, limiting the required time to 6 months minimizes this concern.

## CONCLUSION

Overall, this study evaluating concomitant opioid use with 5αRI decreasing the risk of OUD demonstrated that these medications may play a role in protecting against OUD in men. Prospective studies, including younger and female populations, are needed to confirm this result.

## AUTHOR CONTRIBUTIONS

Abdelrahman G. Tawfik, Tyler Lister, Marco Bortolato, Randall T. Peterson, Ainhoa Gomez‐Lumbreras, and Daniel C. Malone planned the study protocol and study design. Daniel C. Malone acquired the data and conducted the analysis with Tyler Lister and Abdelrahman G. Tawfik. Abdelrahman G. Tawfik, Tyler Lister, and Ainhoa Gomez‐Lumbreras drafted the initial article with all authors reviewing and adding important and critical content. All authors interpreted the data.

## CONFLICT OF INTEREST STATEMENT

The authors declare no conflicts of interest. The authors alone are responsible for the content and writing of this paper.

## ETHICS STATEMENT

This study was approved by the University of Utah Institutional Review Board.

## Supporting information

Supplemental 05‐05‐25.

## Data Availability

The data that support the findings of this study are available from the Department of Health and Human Services ‐ Centers for Medicaid and Medicare (CMS). Restrictions apply to the availability of these data, which were used under license for this study. Data are available from the authors with the permission of CMS.
