# Supplementary material for: An exploratory analysis of 5‐alpha reductase inhibitors and risk of opioid use disorder among male Medicare beneficiaries receiving prescription opioid medications
Source: Am J Addict. 2025 Jul 13;35(1):46–52. doi: 10.1111/ajad.70069 (PMC12747702; doi:10.1111/ajad.70069)
Supplement: Supplementary file 1 — Supplemental 05‐05‐25. [file AJAD-35-46-s001.docx]

**Table S1. Distribution of Patients by Number of Unique Opioid ICD-10 Codes**

| **Group** | **Frequency** | **Percent** | **Cumulative Frequency** | **Cumulative Percent** |
| --- | --- | --- | --- | --- |
| 1 Code | 499 | 85.74% | 499 | 85.74% |
| 2 Codes | 57 | 9.79% | 556 | 95.53% |
| 3 Codes | 20 | 3.44% | 576 | 98.97% |
| >3 Codes | 6 | 1.03% | 582 | 100.00% |

**Table S2. Frequency of Individual F11 ICD-10 Codes Among 582 Patients**
(*Each code counted only once per patient*)

| **ICD-10 Code** | **Description** | **Frequency** | **Percent** |
| --- | --- | --- | --- |
| F11.20 | Opioid dependence, uncomplicated | 352 | 50.5% |
| F11.90 | Opioid use, uncomplicated | 145 | 20.8% |
| F11.10 | Opioid abuse, uncomplicated | 69 | 9.9% |
| F11.23, F11.21, F11.29, etc. | Other F11 subtypes | <5% each | -- |
